# Ecliptasaponin A attenuates renal fibrosis by regulating the extracellular matrix of renal tubular cells

**DOI:** 10.1007/s11626-023-00803-0

**Published:** 2023-10-13

**Authors:** Xiaomin Li, Wenhui Dong, Yanlin Yang, Shijing Ren, Xiangyu Wang, Meina Zou, Wen Lu, Lerong Liu, Yaoming Xue

**Affiliations:** 1 Department of Endocrinology and Metabolism, Nanfang Hospital, Southern Medical University, Guangzhou, China; 2 Department of Endocrinology and Metabolism, The Second Affiliated Hospital of Nanchang University, Nanchang, China

**Keywords:** Renal fibrosis, Extracellular matrix, Ecliptasaponin A, MMP13, Diabetic kidney disease

## Abstract

Renal fibrosis is the most common manifestation of end-stage renal disease (ESRD), including diabetic kidney disease (DKD), but there is no effective treatment in renal fibrosis. Natural products are a rich source of clinical drug research and have been used in the clinical research of various diseases. In this study, we searched for traditional Chinese medicine monomers that attenuate fibrosis and assessed their effect on the fibrosis marker connective tissue growth factor (CTGF) in cells which we found ecliptasaponin A. Subsequently, we evaluated the effect of ecliptasaponin A on renal fibrosis in the classic renal fibrosis unilateral ureteral obstruction (UUO) mouse model and found that ecliptasaponin A could reduce the renal collagen fiber deposition and renal extracellular matrix (ECM) protein expression in UUO mice. In vitro, ecliptasaponin A can inhibit ECM protein expression in human kidney-2 (HK-2) cells induced by transforming growth factor-beta1 (TGFβ1). To further clarify the mechanism of ecliptasaponin A in attenuating renal fibrosis, we performed transcriptome sequencing of HK-2 cells treated with TGFβ1 and ecliptasaponin A. The functions and pathways were mainly enriched in the extracellular matrix and TGFβ signalling pathway. Matrix metalloproteinase 10 (MMP10) and matrix metalloproteinase 13 (MMP13) are the main differentially expressed genes in extracellular matrix regulation. Then, we measured MMP10 and MMP13 in the cells and found that ecliptasaponin A had a significant inhibitory effect on MMP13 expression but not on MMP10 expression. Furthermore, we overexpressed MMP13 in HK-2 cells treated with TGFβ1 and found that MMP13 promoted HK-2 cell injury. Our findings suggest that ecliptasaponin A can attenuate renal fibrosis, which may provide a new method for treating renal fibrosis clinically.

**Supplementary Information:**

The online version contains supplementary material available at 10.1007/s11626-023-00803-0.

## Introduction

Diabetic kidney disease (DKD) is the main cause of end-stage renal disease (ESRD), resulting in a heavy disease burden (GBD Chronic Kidney Disease Collaboration 2020). Renal fibrosis is the final pathological manifestation of DKD. There is no effective treatment for renal fibrosis, so it is urgent to find an effective treatment to delay renal fibrosis. At present, the methods used to attenuate renal fibrosis in the clinic are limited and mainly focus on the treatment of etiology, such as hypotension and hypoglycemia. Many new methods targeting the etiological mechanism of fibrosis are also being carried out, such as TGFβ signalling pathway inhibitors, myofibroblast activation inhibitors, and extracellular matrix regulation (Humphreys 2018).

The use of natural compounds as a means of treating diseases has attracted increasing attention. Natural products have always been a rich source of drug research and development. Anticancer drugs such as paclitaxel and vinblastine and antimalarial drugs such as quinine and artemisinin are all found in natural products (Thomford *et al*. 2018). Natural products can also be used in diabetic kidney disease. Ecliptasaponin A (EA) is a triterpenoid molecule compound extracted from the natural plant *Ecliptae herba*. According to the *Chinese Pharmacopoeia*, EA is the aboveground dry part of the composite plant *Eclipta prostrate* L. When the flowers bloom, they can be picked and then dried in the sun for use as medicine. The main effects of EA are nourishing the liver and kidney and cooling blood for hemostasis (Liu *et al*. 2012). Some scholars believe that *Eclipta* has antihepatoma effects by inhibiting the proliferation and promoting the apoptosis of hepatoma cells by inhibiting the PI3K/AKT signalling pathway (Pan *et al*. 2021). EA has been reported to induce apoptosis and autophagy in human lung cancer cells by activating the ASK1/JNK pathway (Han *et al*. 2019). In addition, EA can inhibit the expression of MMP13 in an osteoarthritis model, thereby reducing the expression of related inflammatory factors in the osteoarthritis model (Hong *et al*. 2018). However, the role of EA in renal fibrosis is unknown.

The extracellular matrix (ECM), a highly dynamic noncellular three-dimensional structure that is in a state of continuous regulation and remodelling, exists in all tissues and plays an indispensable role in tissues and organs (You *et al*. 2015). Excessive deposition of ECM can be observed in renal fibrosis. The most important regulatory factors of ECM are matrix metalloproteinases (MMPs), which can be classified as collagenases, gelatinases, stromalysins, matrilysins, membrane type (MT)-MMPs, and other MMPs (Nagase *et al*. 2006). In renal fibrosis, the most important ECM is type I collagen, and collagenase is the MMP that mainly cleaves collagen.

In this study, we confirmed that EA can reduce the production of ECM proteins in unilateral ureteral obstruction (UUO) mice and renal tubular epithelial cells in vitro and in vivo to prevent renal fibrosis. The possible mechanism was enriched in the process of regulation of the extracellular matrix and the main regulatory factor was MMP13. EA can significantly inhibit the expression of MMP13, which can damage renal tubular epithelial cells.

## Materials and methods

### Preparation of Chinese medicine monomers

The 10 selected traditional Chinese medicine monomers were purchased from the China National Standard Material Network (http://www.bzwz-china.com/) and dissolved in methanol in cell experiments. EA for use in the animal and cell research was purchased from Herb Substance Biotechnology Co., Ltd. (Chengdu, China). In animal experiments, EA was dissolved in olive oil (80 mg/kg/d, concentration was selected according to reference (You *et al*. 2015)). In the cell experiment, EA was dissolved in methanol.

### Cell culture and stimulation - Selecting experiment of traditional Chinese medicine monomers

HK-2 cells were starved in low-glucose DMEM (2% FBS) for 24 h, then changed to high-glucose (30 mM) medium (10% FBS), and added to the same concentration of traditional Chinese medicine monomers (ecliptasaponin A, martynoside, astragalus polysaccharides, emodin, rhein, aucubin, betaine, ursolic acid, icariin, geniposide) for 48 h.

### TGFβ1 stimulation

HK-2 cells were starved in low-glucose DMEM (2% FBS) for 24 h and then changed to low-glucose DMEM (10% FBS), and TGFβ1 (10 ng/mL) was added to each well for 48 h.

### EA treatment

After the HK-2 cells were starved for 24 h, the medium was changed to low-glucose DMEM (10% FBS), and TGFβ1 (10 ng/mL) and EA were added to each well of the plate.

### Animals and drug administration

Six-week-old C57BL/6 J mice (*n* = 24) were purchased from Southern Medical University Laboratory Animal Science and Technology Development Co., Ltd. (Guangzhou, China). Throughout the whole experiment, the mice were provided with sufficient food and water. After 1 wk of adaptive feeding, all mice were anesthetized by intraperitoneal injection of 1% pelltobarbitalum natricum (50 mg/kg). The ureters were separated below the left kidney with the animals in the prone position. In the operation group (unilateral ureteral obstruction, UUO), the left ureters were separated and ligated with No. 4 silk thread to complete the obstruction of the left ureters. In the sham group, the left ureter was separated without ligation, and the other operations were the same as those in the UUO group. The mice were divided into three groups: (1) control group: the mice received sham operation treatment and were given solvent (olive oil) by gavage on the second day after the operation; (2) UUO group: the mice underwent the UUO operation, and the solvent (olive oil) was given by gavage on the second day after the operation; (3) UUO + EA group: the mice underwent the UUO operation. On the second day after the operation, the mice were given EA (80 mg/kg/d) by gavage for 10 consecutive d. Ten days after the operation, the mice were killed. The renal capsule was stripped with curved tweezers, and the kidneys were removed on both sides. Filter paper was used to absorb the surface water, and the kidneys were weighed. The kidney tissue was preserved in a − 80℃ refrigerator.

### Histological analysis

The left kidney tissue was fixed in 4% paraformaldehyde, embedded in paraffin, cut into 6-μm-thick sections, stained with HE, PAS, and Masson’s trichrome and observed under a microscope (BX51, Olympus Corporation, Tokyo, Japan). The area positive for Masson staining was calculated with ImageJ.

### Cell counting Kit-8 assay

HK-2 cells were seeded into 96-well plates. After starving the cells for 24 h, the medium was replaced with low-glucose DMEM (10% FBS) and subjected to the following conditions: (1) blank group: cell-free group containing only medium; (2) 0 dosing group: a group containing cells but without EA; (3) EA group: the cells were subjected to different concentrations of EA (5, 10, 15, 20, 25, 30 nM) for 48 h, and three wells were taken from each group to calculate the average value and draw the proliferation curve. After 48 h, the solution was changed to low-glucose DMEM (10% FBS), and 10 μL of CCK-8 solution was added to each well; the plates were incubated at 37°C for 1–4 h, and the absorbance at 450 nm was measured with a microplate reader.

### RNA extraction library construction and sequencing

HK-2 cells in the control group, TGFβ1 (10 ng/mL) group, and TGFβ1 (10 ng/mL) + EA (20 nM) group were cultured for 48 h, and total RNA was extracted with TRIzol reagent (Beijing Dingguo Biotechnology Co., Ltd, Beijing, China.) according to the manufacturer’s instructions. After total RNA was extracted, magnetic beads with oligo (DT) and Poly A were used for A-T base pairing, and mRNA was isolated from total RNA. Then, the mRNA was randomly broken into small fragments of approximately 300 bp by adding a fragment buffer. The cut RNA fragment was then reverse transcribed by reverse transcriptase to obtain cDNA. Then, a base was added to the 3′ end of each chain and connected to the index adapter. The ligation products were amplified by PCR. Finally, we performed 2 × 150-bp paired-end sequencing (PE150) on the Illumina NovaSeq™ 6000 (LC-Bio Technology Co., Ltd., Hangzhou, China) according to the manufacturer’s protocol.

### Bioinformatics analysis - Quality control, comparison, expression analysis, and expression difference analysis of original sequencing data

Conduct sequencing–related quality evaluation on the raw sequencing data of each sample, including (1) base error rate distribution statistics; (2) base content distribution statistics.

Using software: fastp.

1. Base error rate distribution statistics

The sequencing error rate increases with the length of sequenced reads, which is caused by the consumption of chemical reagents during the sequencing process, and is a common feature of Illumina’s high-throughput sequencing platform. In addition, there is also a high sequencing error rate in the positions of the first six bases, and this length is exactly equal to the length of random primers required for reverse transcription during the RNA seq library construction process. The high sequencing error rate of these bases may be due to incomplete binding between random primers and RNA templates.

2. Base content distribution statistics

The distribution of base content is generally used to detect the presence of AT and GC separation phenomena. For RNA seq, in view of the randomness of sequence interruption and the principle of equal G/C and A/T contents, theoretically, the GC-content and AT content in each sequencing cycle are equal (if it is chain specific database building, AT separation and/or GC separation may occur), and the whole sequencing process is basically stable, showing a horizontal line. However, in existing high-throughput sequencing techniques, the 6-bp random primers used for reverse transcription synthesis of cDNA can cause certain preferences in the nucleotide composition of the first few positions, which is a normal fluctuation.

After obtaining clean data (reads), RSEM (http://deweylab.github.io/RSEM) was used for quantitative analysis of genes and transcripts. RSEM can calculate the expression level of genes/transcripts using single- or double-ended sequencing data. RSEM builds a maximum likelihood abundance estimation model based on the maximum expectation algorithm, and considers paired end reads, the length of reads, the length distribution of fragments, quality values, etc., to distinguish which transcripts are different subtypes of the same gene. Raw read counts of gene expression differences among samples (≥ 2) items were analyzed by using the R package DESeq2. If the conditions (FDR < 0.05 and |log2fc|≥ 1.0) were met, the genes were regarded as differentially expressed genes (DEGs).

### Venn diagrams

From the above differentially expressed genes, the genes that were upregulated in the TGFβ1 group, downregulated in the TGFβ1 + EA group, and had significant differences were used as targets to explore the possible potential mechanism of EA. The specific screening conditions were as follows: (1) Genes were upregulated in the TGFβ1 group compared with the control group and downregulated in the TGFβ1 + EA group compared with the TGFβ1 group; (2) the difference in expression was | log2fc |≥ 1.5; (3) the difference was statistically significant, that is, padjust < 0.05.

A Venn diagram (http://bioinformatics.psb.ugent.be/webtools/Venn/) was used to screen out the genes that met the above conditions involved in the possible mechanism of action of EA.

### Protein‒protein interaction (PPI)

A PPI protein network (https://cn.string-db.org/) was used to analyze the mutual intersection genes screened by a Venn diagram and evaluate the interaction relationship between the proteins encoded by these genes.

### Gene ontology (GO) categories and Kyoto encyclopedia of genes and genomes (KEGG)

Gene Ontology (GO) functional enrichment analysis and Kyoto Encyclopedia of Genes and Genomes (KEGG) enrichment analysis were used to analyze and predict the biological functions of target genes. GO (http://www.geneontology.org/) analysis clarified the main biological processes in three aspects: cell composition, molecular function, and biological process. The results are expressed as *p* values. The lower the *p* value is, the more significant the enrichment result.

KEGG (https://www.genome.jp/kegg/) is a database resource that analyzes differentially expressed mRNAs through genetic biology to explore important pathways related to the target genes. The lower the *p* value is, the more significant the enrichment result.

### Real-time qPCR

The gene expression levels of ECM proteins (CTGF, Col-1, Col-3), α-SMA, E-cadherin, vimentin, MMP10, and MMP13 were evaluated by RT-qPCR. Total RNA was extracted from mouse kidney or HK-2 cells using TRIzol reagent (Dingguo, Beijing, China). Primescript RT Master Mix (Yeasen, Shanghai, China) was used to reverse transcribe RNA into cDNA. Quantitative analysis was performed with a qPCR instrument (LC480) using SYBR ® Green Master Mix (Takara, Kyoto, Japan). β-Actin was used as the internal standard for mRNA. The relative expression of each gene was quantified using the 2^−∆∆Ct^ (∆∆Ct = ( Ct_target gene_-Ct_reference gene_)-mean Ct_reference gene_) method. The primer sequences are shown in Supplementary Table 1.

### Western blot

The kidney tissue of mice or HK-2 cells was lysed with RIPA lysis buffer (Beyotime, Beijing, China) containing 0.1% protease inhibitor and 1% phosphatase inhibitor for 10 min and centrifuged at 12,000 rpm for 15 min, and the supernatant was transferred to a new test tube. A BCA Protein Assay Kit (Takara, Kyoto, Japan) was used to assess the protein concentration. A Gel Preparation Kit (Yisheng, Shanghai, China) was used for SDS-PAGE. The isolated proteins were transferred to polyvinylidene fluoride (PVDF) membranes (Millipore) and blocked with primary dilution antibodies overnight at 4°C. Anti-Hsp90 primary antibody (1:1000, mouse, TA-12, ZSGB-BIO, Beijing, China), anti-FN (1:1000; rabbit; A16678; Abclonal, Boston, MA), anti-Col-1 (1:1000, rabbit, A1352, Abclonal, Boston, Massachusetts), anti-Vimentin (1:1000, rabbit, 10,366–1-AP, Proteintech, Rosemont, IL), anti-TGFβ (1:1000, rabbit, A18692, Abclonal), anti-Col-3 (1:1000, rabbit, WL03186, WanleiBio, Shenyang, China), anti-MMP10 (1:1000, rabbit, MAB9101, R&D, Minneapolis, MN), and anti-MMP13 (1:1000, rabbit, A11755, Abclonal) were used as the primary antibodies following incubation with the membranes for 12 h at 4°C. After sufficiently washing the membranes with Tris-buffered saline + 0.1% [v/v] Tween-20 (TBST), the membranes were incubated with the corresponding secondary antibody for 1 h at room temperature and then washed three times with TBST. The protein level was determined by BLT GelView 6000 Pro enhanced chemiluminescence (FDbio, Hangzhou, China).

### Immunofluorescence

HK-2 cells induced by TGFβ1 and EA treatment for 48 h were washed with PBS 3 times. The cells were fixed with 4% paraformaldehyde (PFA) for 15 min, permeabilized with 0.1% Triton X-100 for 20 min, washed with PBS, and blocked with 5% BSA at room temperature for 2 h. Then, the cells were incubated with anti-MMP13 antibody (1:100, rabbit, A11755, Abclonal) overnight at 4°C. The next day, the slides were washed in PBS and incubated with the appropriate secondary antibodies (Proteintech, Rosemont, IL, sa00013-4, 1:200) (Proteintech sa00013-3, 1:200) at room temperature for 1 h. In the last step, the cover glass was washed with a fade-resistant mounting medium with DAPI and mounted on the slide. Images were captured using a fluorescence inverted microscope (Imager D2, Zeiss, Oberkochen, Germany).

### Immunohistochemistry

The preparation of slides was the same as that for histological analysis. EDTA antigen repair solution was used for antigen repair and then cooled at room temperature. After the samples were washed with PBS, 3% hydrogen peroxide solution was used to block the cells for 10 min. MMP13 primary antibody was incubated at 4°C for 12–18 h. The next day, the slides were washed in PBS and incubated with the appropriate secondary antibody at room temperature for 30 min. After washing, DAB was added to the slides and incubated for 1 min. After coloring, the slides were washed, stained with hematoxylin for 3 min and 1% hydrochloric acid alcohol solution for 2 s, and baked in an oven at 60℃ for 10 min. Images were captured using a microscope (BX51, Olympus Corporation, Tokyo, Japan).

### Plasmid construction and cell transfection

The MMP13 overexpression plasmid was constructed by WZBio (Shandong, China). According to the manufacturer’s protocol, 2500-ng plasmid and 6 μL Lipofectamine® 3000 transfection reagent (Invitrogen, Carlsbad, CA) were used to transfect cells. RNA and protein expression levels were detected 48 h after transfection, and each experiment was repeated at least three times. The overexpression efficiency of MMP13 was verified by RT-qPCR.

### Statistical analysis

GraphPad Prism 8.0.1 was used for data analysis and mapping, and the data are expressed as the mean ± standard deviation (‾χ ± SD). A two independent-sample *t* test was used to compare two groups of measurement data that conformed to a normal distribution, and one-way ANOVA was used to compare the mean between multiple groups. When *p* < 0.05, the difference was considered statistically significant.

## Results

### EA inhibits the expression of the fibrosis marker CTGF

**Figure 1.**
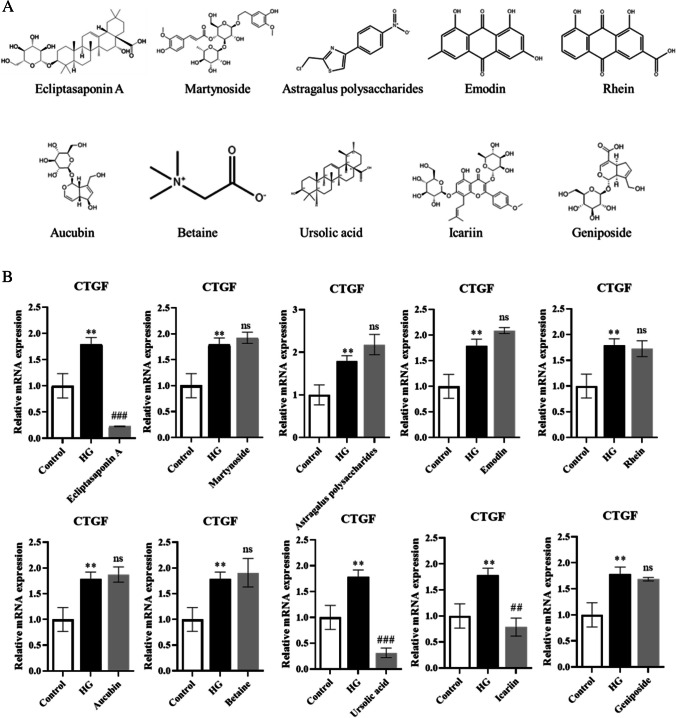
EA inhibits the expression of fibrosis marker CTGF. (*A*) The chemical mechanism of 10 selected Chinese medicine monomers. (*B*) The mRNA expression of fibrosis marker CTGF effected by the 10 kinds of traditional Chinese medicine monomers. Values are the means ± SD. **p < 0.05 vs. control group; ##*p* < 0.01, ###*p* < 0.001 vs. HG group. Non-significant differences vs HG group are denoted by *ns.*

To explore the natural products that can attenuate renal fibrosis, we searched the traditional Chinese medicine monomers which were recorded on the Chinese standard material network (with high purity) with the effect of attenuating fibrosis on PubMed. We obtained 10 traditional Chinese medicine monomers, including ecliptasaponin A, martynoside, astragalus polysaccharides, emodin, rhein, aucubin, betaine, ursolic acid, icariin, and geniposide (Fig. 1*A*). Then, we inuced HK-2 cells with high-glucose (HG) and treated them with different traditional Chinese medicine monomers. By measuring the mRNA expression level of the fibrosis marker CTGF, we preliminarily selected the appropriate traditional Chinese medicine monomers for follow-up research. The results showed that among the 10 traditional Chinese medicine monomers, EA had the most obvious inhibition of the fibrosis marker CTGF (Fig. 1*B*), so we chose EA for the follow-up study.

### EA ameliorates the obstructive kidney weight ratio in UUO mice

**Figure 2.**
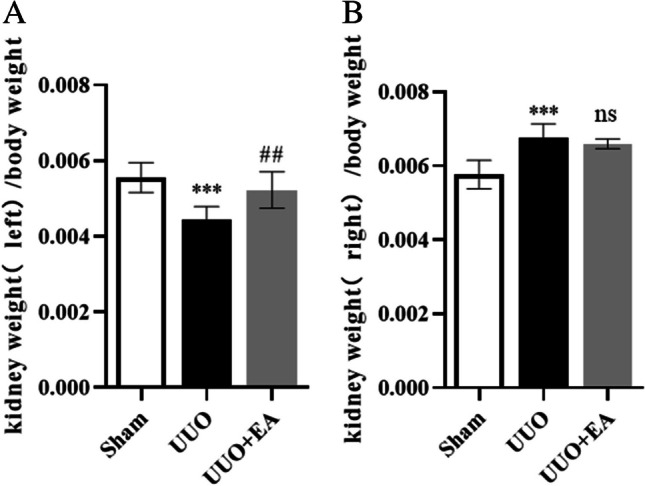
Kidney weight ratio among mice in each group. (*A*) Left kidney weight/body weight of mice. (*B*) Right kidney weight/body weight of mice. Values are the means ± SD. ***p < 0.001 vs. control group; ##*p* < 0.01 vs. UUO group. Non-significant differences vs UUO group are denoted by *ns.*

We used UUO mice for simulation of renal fibrosis and evaluation of the effect of EA on markers related to renal fibrosis in mice. We divided the mice into three groups: sham, UUO, and UUO + EA. The UUO + EA group was treated with EA (80 mg/kg/d) by gavage, and the sham and UUO mice were treated with the same volume of solvent. After 10 d, the mice were killed, the kidney tissues were collected, and changes in the kidney weight ratio were detected. We found that compared with that in the sham group, the left (obstructive side) kidney weight/body weight ratio of UUO group mice decreased significantly, and the right kidney weight/body weight ratio increased significantly, suggesting that UUO led to the loss of renal parenchyma in the obstructed side in the mice and the compensatory function of the contralateral kidney. After UUO mice were treated with EA, the renal weight of the left side was significantly higher than that in the UUO group, and the renal weight ratio of the contralateral side was changed little between the UUO + EA group and the UUO group (Fig. 2), indicating that EA may reduce the loss of renal parenchyma of the obstructed side kidney caused by UUO, suggesting that EA may have a certain renal protective effect.

### EA attenuates histological damage and collagen deposition in the kidneys of UUO mice

**Figure 3.**
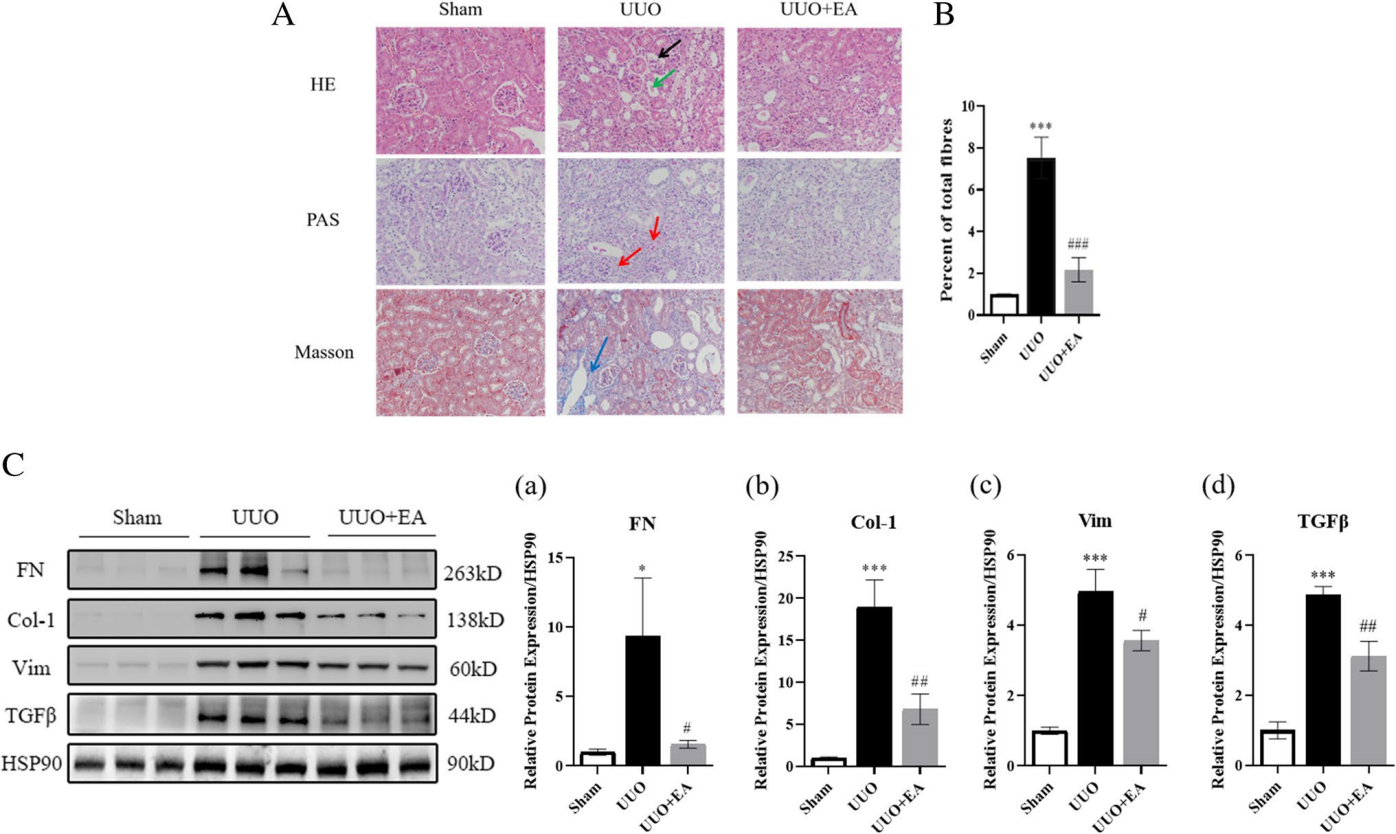
Kidney pathology and ECM protein deposition in mice. (*A*) HE, PAS, and Masson staining of mice renal cortex (× 400),  glomerular atrophy,  renal tubular dilatation,  thickening of renal tubular and glomerular basement membrane,  collagen fiber deposition. (*B*) Quantification of collagen fibers of Masson stained in mouse renal cortex. (*C*) Expression of extracellular matrix protein in mice of each group and quantification of extracellular matrix protein like FN (*a*), collagen Ι (*b*), vimentin (*c*), and TGFβ (*d*). Values are the means ± SD. *p < 0.05, ***p < 0.001 vs. control group; #*p* < 0.05, ##*p* < 0.01, ### *p* < 0.001, vs. UUO group.

Next, we performed HE, PAS, and Masson staining on the kidneys of the mice to evaluate histological damage and collagen fiber deposition in the kidney. Compared with that in the sham group, the kidney structure of UUO mice was disordered, the glomerulus was atrophic, mesangial dilatation and basement membrane thickening were observed in the glomerulus and renal tubular areas, renal tubules were dilated, and a large number of collagen fibers were deposited in the renal tubulointerstitium. In the UUO + EA group, the increase in the tubulointerstitium was less than that in the UUO group, and the deposition of blue collagen fibers was reduced (Fig. 3*A* and *B*). It is suggested that EA can reduce the deposition of collagen fibers in the renal interstitium of the obstructed side of UUO mice. Furthermore, we extracted total protein from the mouse renal cortex for Western blotting and measured the ECM protein level. EA reduced the expression of ECM proteins in the renal tissues of UUO mice (Fig. 3*C*).

### EA reduces the expression of ECM proteins in HK-2 cells induced by TGFβ1

**Figure 4.**
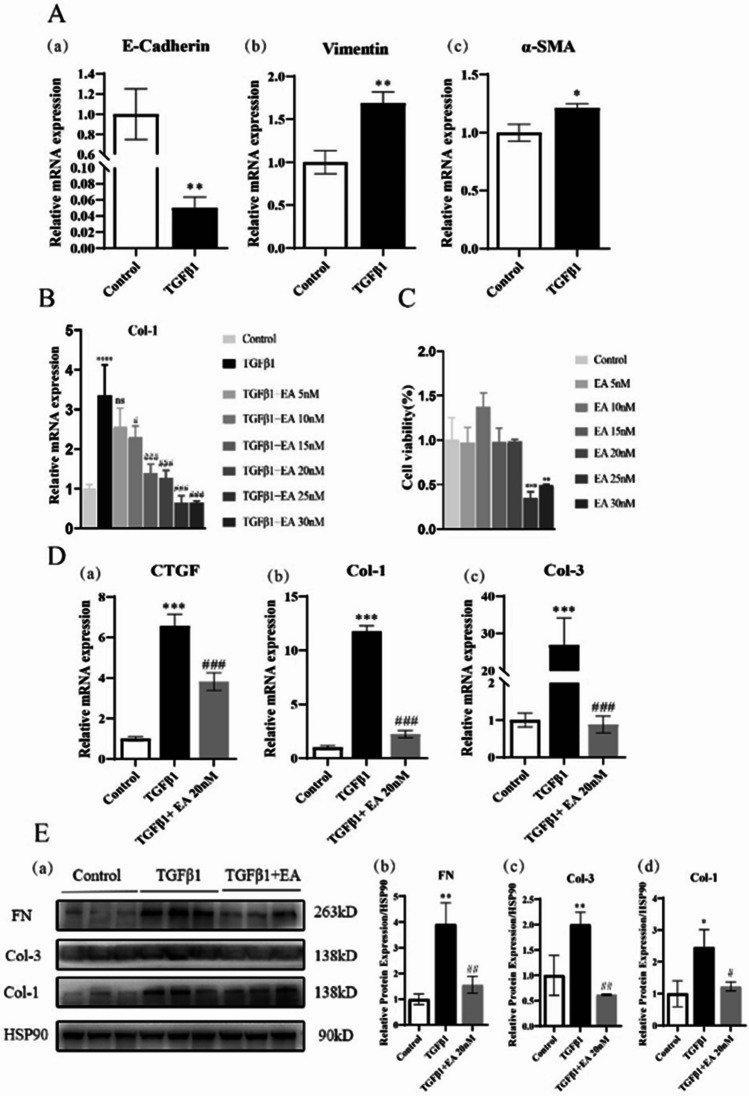
Effect of EA on ECM in HK-2 cells. (*A*) Expression of EMT-related gene mRNA E-cadherin (*a*), vimentin (*b*), α-SMA (*c*), in HK-2 cells. (*B*) The mRNA level of Col-1 in HK-2 cells under different concentrations of EA. (*C*) Cell activity of HK-2 cells at different concentrations of EA. (*D*) mRNA expression of ECM protein CTGF (*a*), collagen Ι (*b*), collagen Ш (*c*), in HK-2 cells. (*E*) Protein expression of ECM (*a*) in HK-2 cells and quantification of extracellular matrix protein FN (*b*), collagen Ш (*c*), collagen Ι (*d*). Values are the means ± SD. *p < 0.05, **p < 0.01, ***p < 0.001 vs. control group; #*p* < 0.05, ##*p* < 0.01, ### *p* < 0.001, vs. TGFβ1 group.

Then, we tested the effect of EA in vitro. The ECM is an important manifestation of renal fibrosis. We used TGFβ1 to induce EMT in HK-2 cells, characterized by the decreasing of E-cadherin and increasing of the vimentin and α-SMA (Fig. 4*A*). Then, we explored the optimal concentration of EA in vitro. EA decreased the expression of type I collagen in a concentration-dependent manner (from 5 to 25 nM). When the concentration of EA was greater than 25 nM, EA inhibited cell activity. According to the results of EA on collagen I and its influence on cell activity, we subsequently selected EA to treat cells at 20 nM (Fig. 4*B* and *C*). We also tested the expression of ECM protein such as FN, Col-1, Col-3, and CTGF in HK-2 cells, and found that EA also inhibited the gene and protein expression of ECM proteins induced by TGFβ1 in HK-2 cells (Fig. 4*D* and *E*).

### Possible mechanism of EA in attenuating renal fibrosis

**Figure 5.**
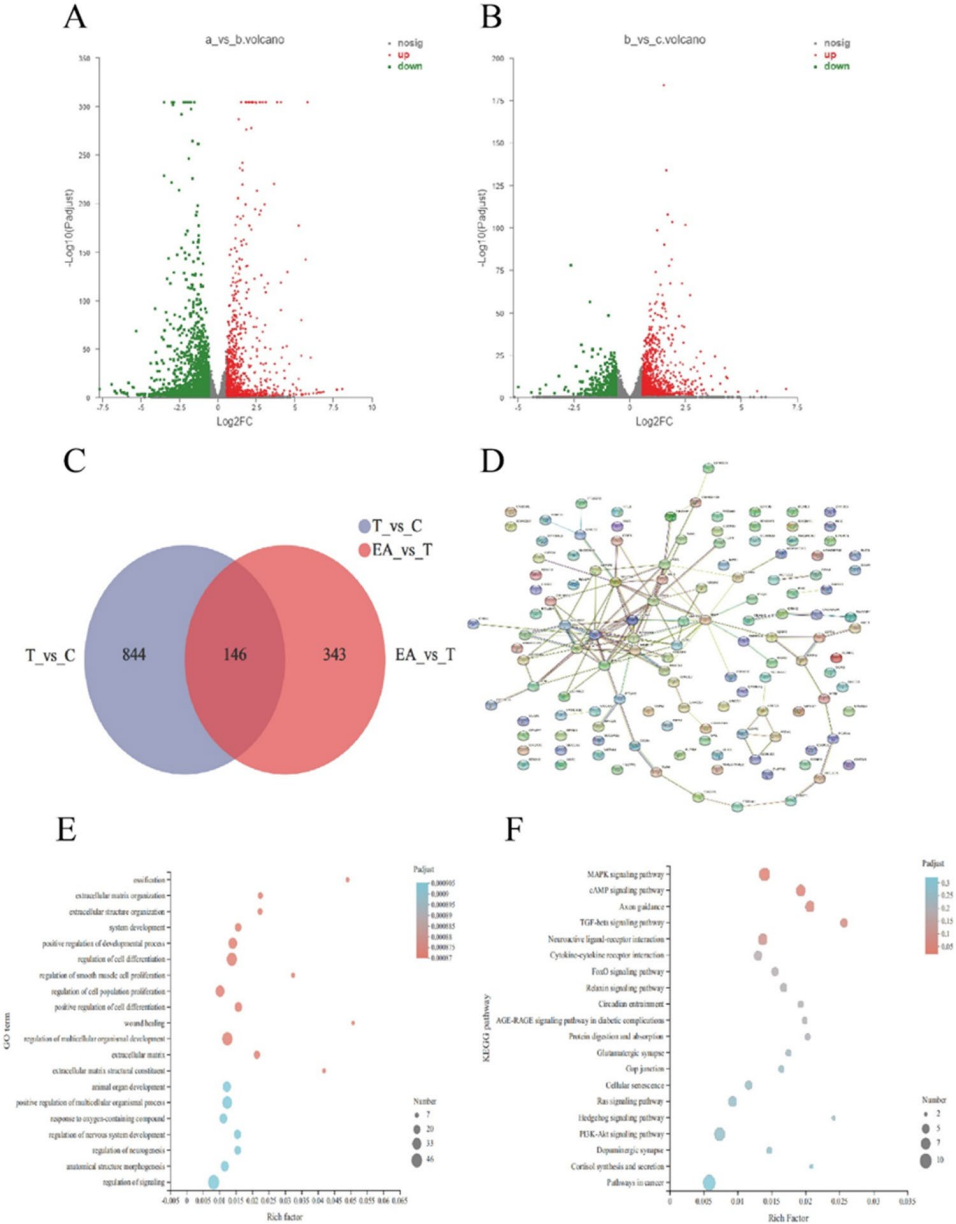
The possible mechanism of EA attenuating fibrosis in HK-2 cells. (*A*) Difference genes in TGFβ1 compared with control group. (*B*) Difference genes in TGFβ1 + EA compared with TGFβ1 group. (*C*) Venn diagram analysis of the difference genes. (*D*) PPI analysis of the intersected difference genes. (*E*) GO enrichment of the intersected difference genes. (*F*) KEGG enrichment of the intersected difference genes. *c*: Control group, *T*: TGFβ1 (10 ng/mL), *EA*: TGFβ1 (10 ng/mL) + EA (20 nM) group.

To explore the possible mechanism of EA in attenuating renal fibrosis, we extracted total RNA from the control group, TGFβ1 group, and TGFβ1 + EA group cells and sequenced the transcriptome. Through the analysis of sequencing data, we selected the DEGs with differences in expression of |log2fc|≥ 1.5, and the difference was found to be statistically significant, that is, padjust < 0.05. We found that 990 differentially expressed genes were upregulated and 1683 differentially expressed genes were downregulated in the TGFβ1 group compared with the control group (Fig. 5*A* and *B*). Moreover, 946 differentially expressed genes were upregulated and 489 differentially expressed genes were downregulated in the TGFβ1 + EA group compared with the TGFβ1 group. From these differentially expressed genes, we selected those that were upregulated in the TGFβ1 group and downregulated in the TGFβ1 + EA group for Venn diagram analysis. We found 146 differentially expressed genes that intersected with each other (Fig. 5*C*). Through PPI network diagram analysis of the 146 differentially expressed genes, a close interaction was found between type I, III, and V collagen and the matrix metalloproteinases MMP10 and MMP13 (Fig. 5*D*). Subsequently, we performed GO function and KEGG pathway enrichment analyses on these 146 differentially expressed genes, which suggested that extracellular matrix, regulation of cell differentiation, regulation of development, and the TGFβ signalling pathway are related to the mechanism by which EA attenuates fibrosis (Fig. 5*E* and *F*). MMPs play a major role in the regulation of the extracellular matrix. Among the differentially expressed genes, MMP10 and MMP13 were the differentially expressed MMPs, suggesting that EA may act through MMP10 or MMP13.

### EA inhibits the expression of MMP13

**Figure 6.**
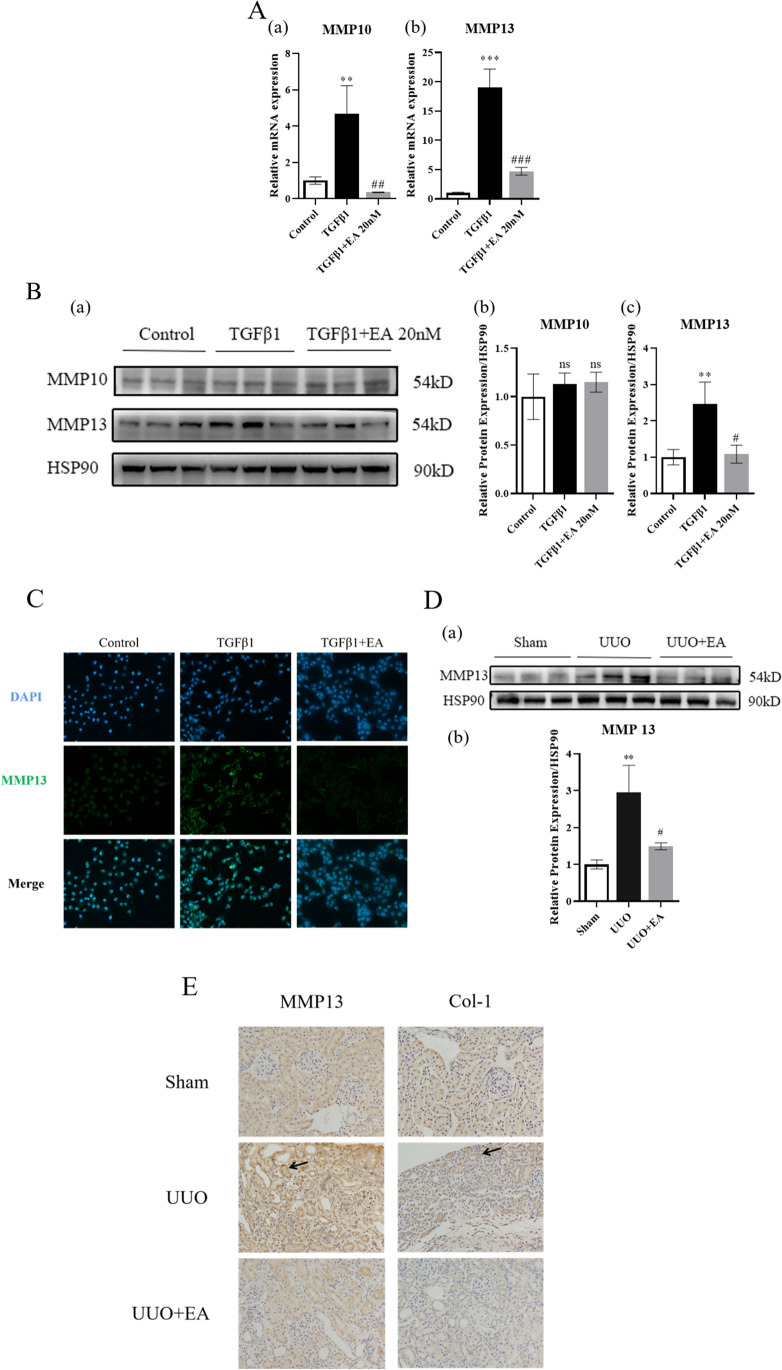
Expression of MMP10 and MMP13 in HK-2 cells and mouse kidney. (*A*) The mRNA level of MMP10 and MMP13 in HK-2 cells. (*B*) The protein expression of MMP10 and MMP13 (*a*) and quantification of MMP10 (*b*) and MMP13 (*c*) protein in HK-2 cells. (*C*) The fluorescence level of MMP13 in HK-2 cells. (*D*) The protein expression of MMP13 (*a*) quantification of MMP13 (*b*) protein in mouse kidney. (*E*) The immunohistochemistry level of MMP13 and Col-1 in mouse kidney. **p < 0.01, ***p < 0.001 vs. control or sham group; #*p* < 0.05, ##*p* < 0.01, ###*p* < 0.001, vs. TGFβ1 or UUO group.

We detected the expression of gene and protein expression of MMP10 and MMP13 in HK-2 cells and found that at the gene level EA significantly inhibited the mRNA expression of MMP10 and MMP13.While at the protein level, EA significantly inhibited the level of MMP13, but its effect on MMP10 was not obvious (Fig. 6*A* and *B*). Therefore, we selected MMP13 for further study. Immunofluorescence showed that EA inhibited the expression of MMP13 (Fig. 6*C*). At the same time, the immunohistochemistry showed that in the kidneys of UUO mice, EA inhibited the protein expression of MMP13 (Fig. 6*D* and *E*).

### MMP13 promotes renal tubular epithelial cell injury

**Figure 7.**
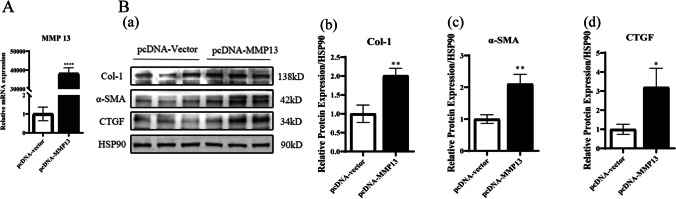
Overexpression of MMP13. (*A*) Overexpression efficiency of MMP13 in HK-2 cells. (*B*) Protein levels of fibrosis markers and quantification of fibrosis markers protein Col-1 (*b*), α-SMA (*c*), and CTGF (*d*) in HK-2 cells after overexpression of MMP13.

Finally, we overexpressed MMP13 in HK-2 cells to determine its effect on renal tubular epithelial cells (Fig. 7*A*). After RT-qPCR was used to verify the overexpression efficiency, Western blotting was used to detect the fibrosis products Col-1, α-SMA, and CTGF. It was found that overexpression of MMP13 could promote the expression of these proteins, suggesting that MMP13 could promote renal tubular epithelial cell injury (Fig. 7*B*). Inhibiting the high expression of MMP13 in the pathological state may be a target for improving renal fibrosis, and EA can inhibit MMP13.

## Discussion

Unilateral ureteral obstruction (UUO) is a classic model of renal fibrosis and can be established in a short time (Chevalier *et al*. 2009). In the UUO model, the left ureter was ligated with silk thread, and the kidney on the ureteral side was ligated, which was called the obstructed kidney, while the kidney on the nonligated side (opposite side) was called the nonobstructed kidney (Martínez-Klimova *et al*. 2019). The pathophysiological process after UUO includes an increase in hydrostatic pressure caused by urine retention triggering the activation of the renin-angiotensin system (RAS). Additionally, angiotensin II activates nicotinamide adenine dinucleotide phosphate (NADPH) oxidase (NOX) to produce reactive oxygen species (ROS). The production of ROS leads to oxidative stress and inflammation and finally to cell death through cell apoptosis or necrosis (Martínez-Klimova *et al*. 2019). These mechanisms also induce resident fibroblasts in the kidney to activate myofibroblasts, replace lost epithelial cells with extracellular matrix (ECM), and promote progressive fibrosis (Xia *et al*. 2018). Our study showed that the renal tissue structure of UUO mice was disordered, glomerular atrophy was observed, renal tubules were dilated, there was clear deposition of collagen fibers in the renal tubulointerstitium, and ECM protein accumulated in the kidney.

Studies have shown that ECM components mainly come from myofibroblasts. In the process of renal fibrosis, there are still many disputes about the origin of myofibroblasts. Many studies have concentrated on showing that renal tubular epithelial cells can be a source of myofibroblasts through the tubular epithelial-mesenchymal transition (EMT) (Loeffler & Wolf 2015). In addition, endothelial cells, hematopoietic cells, and mesenchymal cells are potential sources of myofibroblasts. TGFβ1 is the most effective inducer for initiating and completing EMT in renal tubular epithelial cells (Li & Bertram 2010; Hu *et al*. 2015). Therefore, in the in vitro model, we used TGFβ1 to induce EMT of HK-2 cells.

The ECM is present in all organisms and is in a state of continuous remodelling. ECM remodelling is a very complex process that requires cells to continuously carry out biological activities such as synthesis, degradation, recombination, and chemical modification. To maintain tissue homeostasis, these remodelling processes need very strict regulation (Lu *et al*. 2011). Especially in response to injury, strict regulation of the ECM is particularly important. Dysfunctional ECM remodelling is closely related to pathological conditions and may aggravate disease progression. The ECM is composed of approximately 300 proteins, including collagen, proteoglycans (PG), and glycoproteins (Hynes & Naba 2012). Collagen is the main structural protein of the ECM. In renal fibrosis, type I collagen is the main component of the ECM. The steady state of the ECM plays an important role in maintaining the normal function of tissues. The increased degradation of ECM can lead to the destruction of tissues. For example, abnormally high expression levels of cardiac-specific MMP1 lead to the loss of collagen and a decline in cardiac contractility, leading to cardiomyopathy (Kim *et al*. 2000). Excessive deposition of ECM leads to pathological fibrosis. For example, ECM deposition in the kidney is a typical manifestation of renal pathology in DKD. In DKD, ECM deposition causes glomerular and tubular basement membrane thickening, followed by mesangial dilatation, glomerulosclerosis, and tubulointerstitial fibrosis (Garcia-Fernandez *et al*. 2020). This shows that the strict balance regulation of ECM plays an important role in DKD, and the imbalance of this regulation is an important factor that aggravates the progression of DKD. Reducing the imbalance of regulation may be a means for delaying the progression of DKD.

The exact pathogenesis of renal fibrosis is still unclear. Renal fibrosis is a late manifestation of CKD of all causes, including DKD, and is an important factor in the progression to ESRD. An increasing number of people are focusing on exploring new therapies to attenuate fibrosis. Currently, clinically approved drugs for renal fibrosis include RAS blockers (Ruiz-Ortega *et al*. 2020), the third-generation mineralocorticoid receptor antagonist finerenone (Grune *et al*. 2018) and SGLT2 inhibitors (canagliflozin and dapagliflozin). Pentoxifylline, which is not approved for renal protection, has been proven to have a protective effect on renal function (Navarro-González *et al*. 2018). Other drugs, such as pirfenidone, which targets TGFβ1, and lademirsen, which targets miR21, and pulmonary fibrosis drugs, such as nintedanib, PRM-151, epigallocatechin gallate, ziritaxestat, trametinib (MEK inhibitor), and anti–IL-11 antibody, are under study (Ruiz-Ortega *et al*. 2022). More drugs are needed to more effectively delay renal fibrosis.

The use of natural compounds as a means of treating diseases has attracted increasing attention. Natural products have always been a rich source of drug research and development. Compounds extracted from natural products have been studied for a variety of diseases and can achieve good curative effects.For example, Korean red ginseng can attenuate diabetic kidney disease by reducing renal inflammation and fibrosis (Karunasagara *et al*. 2020). Icaritin, the main component of flavonoids isolated from herbaceous plants, can significantly alleviate early renal injury in DKD rats by regulating the expression of TGFβ and collagen IV (Qi *et al*. 2011). Acteoside can inhibit the NADPH/oxidase TGF-β/Smad signalling pathway to attenuate kidney injury in db/db mice (Wang *et al*. 2021).

We searched for traditional Chinese medicine monomers that can attenuate fibrosis and have been recorded on the Chinese reference material website (with high purity). We obtained 10 traditional Chinese medicine monomers and used these monomers to treat HK-2 cells induced with high glucose levels. We found that EA has a significant inhibitory effect on the fibrosis marker CTGF. EA is a triterpenoid small molecule compound extracted from the natural plant *Ecliptae herba*. Some scholars believe that *Eclipta* has an antihepatoma effect, which can inhibit the proliferation and promote the apoptosis of hepatoma cells. This effect is mediated by inhibiting the PI3K/AKT signalling pathway (Pan *et al*. 2021). Professor Renhe Lv, a well-known master of Chinese traditional medicine, has used *Eclipta* and *Ligustrum lucidumin* in the treatment of diabetic kidney disease and diabetic retinopathy. Some researchers have used network pharmacology methods to explore the possible mechanisms of *Eclipta* and *Ligustrum lucidumin* and found that their effective components may regulate the body’s oxidative stress, inflammation, glucose, and lipid metabolism through AGE-RAGE, TNF, IL-17, and other signalling pathways. Animal experiments show that *Eclipta* can improve liver lipid metabolism in high-fat diet-fed mice (Zhao *et al*. 2015), prevent osteoporosis in mice (Zhao *et al*. 2019), delay ageing (Xia *et al*. 2019), and protect the liver (Luo *et al*. 2018). A variety of active chemical components, such as flavonoids, triterpenoid saponins, coumarins, thiophenes, and phenolic acids, are present in *Eclipta*, and EA is the main triterpenoid compound of *Eclipta*. EA has been reported to induce apoptosis by activating the ASK1/JNK pathway and autophagy in human lung cancer cells (Han *et al*. 2019). EA can also promote autophagy and apoptosis of hepatoma cells (Liu *et al*. 2012). In addition, EA can inhibit the expression of MMP13 in an osteoarthritis model, thereby reducing the expression of related inflammatory factors in the osteoarthritis model (Hong *et al*. 2018). Studies have shown that intragastric administration of EA can reduce bleomycin-induced pulmonary fibrosis in mice (You *et al*. 2015). The results showed that EA had antitumor, anti-inflammatory, and antifibrotic effects. It can attenuate pulmonary fibrosis to some extent, but the effect of EA on renal fibrosis has not been described. Our study showed that EA can reduce extracellular matrix deposition in the kidneys of UUO mice and attenuate renal fibrosis. In addition, MMP13 promotes the expression of ECM proteins in renal tubular epithelial cells, and EA can inhibit the expression of MMP13 in these cells. Therefore, we believe that EA can attenuate renal fibrosis by regulating the extracellular matrix of renal tubular epithelial cells, and its mechanism may be partially mediated by inhibiting the high expression of MMP13. Our study also has limitations. First, how EA regulates MMP13 needs to be further clarified. Second, the toxicity of EA in animals should be evaluated. Third, the efficacy of EA in the treatment of renal fibrosis still needs to be confirmed by a multicenter, double-blind, randomized controlled clinical study.

### Supplementary Information

Below is the link to the electronic supplementary material.

Supplementary file1 (XLSX 9 KB)

## Data Availability

The data that supports the findings of this study are available in the supplementary material of this article. If there are more requirements, the data are available from the corresponding author upon reasonable request.
